# Visual Multimedia Intelligent Computing System for Seismic Performance of Bridge Structure Based on Object-Oriented Technology

**DOI:** 10.1155/2022/8250649

**Published:** 2022-06-02

**Authors:** Xinliang Zheng, Yi Xie

**Affiliations:** Beihua University, Jilin 132013, China

## Abstract

In order to further promote the standardization of seismic performance design of bridge structures, one must ensure the quality of bridge design and improve design efficiency. First, based on the basic principle of the probabilistic pushover method, the randomness of the structural pushover curve is attributed to the randomness of plastic hinges. Second, the visualization of seismic performance of bridge structures based on the object-oriented technology is adopted. So that the randomness of seismic action can be considered in the analysis of seismic performance. Finally, the limit state equation of the seismic performance of the structure is used to evaluate the reliability of the seismic performance of the bridge in each limit state conveniently. The results show that the failure probability of the structure in each limit state is less than 0.5 under different basic accelerations from 0 to 0.4 g. The software method is simple in calculation and has a strong adaptability, avoiding the difficulty of seismic reliability analysis caused by large-scale simulation and large amount of calculation of traditional structures. Therefore, this method can be easily applied to the probabilistic analysis of seismic behavior of bridge structures under large earthquakes without evaporative deformation failure criterion.

## 1. Introduction

With the development of economy and society, the number of Bridges in China has been increasing rapidly. According to statistics, by the end of 2020, China had built about 833,000 roads and bridges, ranking first in the world. Large-scale bridge construction costs billions or even tens of billions of dollars, and its life span is usually more than 100 years. According to data released by the American Society of Civil Engineers, more than 40 percent of Bridges in the United States were 50 years old or older in 2017, requiring an estimated $123 billion for repairs and enhancements. Bridge maintenance management, the management and the combination of the modern science and technology, engineering, management, and statistics of different subjects such as science and computing science, is a collection of actual bridge condition assessment, degradation prediction, and maintenance scheme selection of integrated information software; its goal is to achieve the total life cycle cost structure and the balance between the maintenance effect through distribution using limited resources. Currently, the construction of infrastructure in our country is gradually from “large-scale construction” to “paying equal attention to construction and custody” transition; the bridge as an important part of national infrastructure and the hub of road software in its custody and maintenance plays an important role in urban sustainable development, with the best cost-effective operation and maintenance strategy also promote social and economic activities of the core elements [1].

The earliest bridge structure analysis software adopts the process-oriented method, that is, for a certain analysis object, programming is realized through flow and sequence. Process-oriented software computing requires software calculators to write and be familiar with the internal details of the software. The programming mode is just like the agricultural economy in the past, where every living thing and production means are produced by themselves. In the structured method proposed later, the analysis object is decomposed into modules according to functions, and the specific details are realized by functional modules. Each functional module can be written separately by many software calculators, and the analysis process is finally completed through function calls. The division of labor and cooperation of structured software computing reflects the spirit of socialized mass production. Although the structure of software computing improves the development efficiency and maintainability of the software to some extent, the reusability and extensibility of the software still do not improve much. This is mainly caused by two reasons. First of all, the structured method adopts the guiding ideology of “task-oriented.” If a new “task” is added to the software calculated for a particular task, the software modification will involve all aspects of the original software, thus resulting in low software development efficiency. Second, data and functional modules are separated in a structured software. When new functional modules are added to the software, new data structures need to be added. The stronger the function of the module is, the larger the amount of data is, and the more complex the coordination relationship between the two will be. Data structure for a problem, because of its particularity, does not modify very hard commonly used for similar problem, separating the data, and function module makes the function of the module, within the scope of the restrictions in smaller software member can only use a few small parts in software assembly, while avoiding the understanding of each parts and components manufacturing process, but we need to be concerned about the software structure level that is relatively low, which requires the software member in all aspects of the software are more professional the emergence of the object-oriented technology overcome the disadvantages of structured software calculation, for the function module of integrated, reusable, extensible, and software expansion flexibility provided favorable conditions. The program software based on object orientation is beneficial to the cooperation among units and the modification of the software. With the emergence of new computing concepts and new construction techniques in bridge engineering, the software will be optimized and modified and the emergence of object-oriented technology conforms to this development. Sun et al., in order to reveal the dynamic response characteristics, dynamic response of induction mechanism, and macroscopic and microscopic characteristics, covered bridge and tunnel structure under the seismic action of soft or weak surrounding rocks of the key components of the cumulative damage effect and the dynamic behavior of disasters, shaking the table test model method for the complex structure in high speed railway. According to the similarity theory and model test technology combined with the existing engineering examples, the dynamic characteristics of the tunnel and the influence motion parameters of the bridge structure caused by different grounds are considered. Hou proposed at the same time that the characteristics of the shaking table model test method for bridge and tunnel covered structure are analyzed, and compared with other research methods, and the test platform and test method are introduced [2]. The preliminary test results show that this method can simulate the seismic dynamic response, damage and catastrophic behavior of bridge, and tunnel covered structures under weak surrounding rock conditions. This is an effective and operable test method, which can be used as a reference for similar tests in the future [3]. Guo et al. proposed a dependency empirical vulnerability modeling method. In the proposed model, the model parameters are associated with scour depth in terms of quadratic polynomials, which provides great flexibility to consider the complex effects of bridge scour. Numerical analysis of pile foundation based on a simple double-span bridge model shows the effectiveness and accuracy of the proposed method. The fragile surface produced in this work can be used to evaluate the seismic elasticity of a wash bridge under seismic loads [4].

On the basis of the current research, the object-oriented intelligent visual calculation software for the seismic performance of bridge structures proposed in this paper can not only analyze the seismic performance reliability based on the strength failure but also analyze the inelastic seismic performance reliability of the structures under large earthquakes conveniently. Using this method, the failure probability of the bridge structure under the seismic level can be calculated directly, and if the economic losses under different failure states of the bridge structure are given, the expected economic losses caused by seismic disasters can also be estimated during the design life [5].

## 2. Research Methods

### 2.1. Analysis Method and Improvement of Seismic Performance of Bridge Structures

#### 2.1.1. Static Structure Analysis

**Figure 1:**
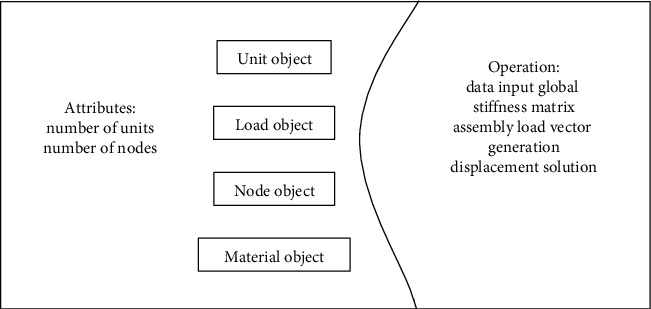
Composition of the overall structure object.

Static structures describe objects, classes, and relationships between classes. According to the static bridge structure analysis method, the structure is divided into the form as shown in Figure 1. The whole structure is described by an integral structure class, which can include attribute data such as component unit object, load object, node object, material object, and multiple operations [5].

**Figure 2:**
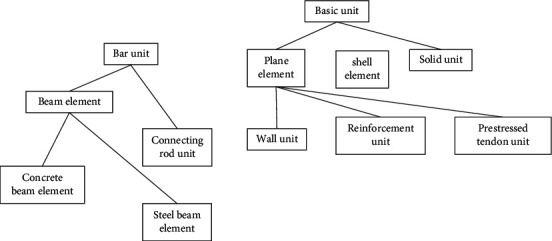
Hierarchy of unit classes.

On the basis of determining the object and the object class, the relationship between the object class must be determined. The relationships between object classes are as follows:

*(1) Hierarchical relationship*. In the process of analysis, classes are organized into different levels according to their generality and individuality. High-level class expression generality forms the parent class; low-level classes express personalities and form subclasses. Subclasses obtain the properties and operations of their parent class through inheritance mechanisms.  Figure 2 shows the hierarchical relationship of element classes of bridge structure components. The basic element at the highest level includes common elements, such as node number and node number. At the second level, according to the geometric shape of the element, it is divided into rod element, plane element, plate and shell element, solid element, etc. In the rod element class, it is divided into the beam element and connecting rod element according to different stress characteristics of components. Then, according to different components or mechanical characteristics, it is divided into concrete and steel beams. Hierarchical classification can have a variety of standard subdivision, and it can also be based on the basic composition of materials to divide the hierarchy. There are also units that need to inherit different superclasses depending on the application. According to the plane element description, it is divided into components such as ordinary reinforcement and prestressed reinforcement unit and is derived from the plane element; when used as a rod element, it shall be described as a rod element and it is derived from the rod element [6–9].

*(2) Aggregation relationship*. Aggregate relationships are combinatorial constructional relationships between objects. The combination of objects is carried out according to a certain hierarchical relationship. The high-level object is the container object, which is called the aggregate object. The lower-level objects are the inclusion objects called constituent objects. Aggregate objects implement their own operations by the operations that make up the objects. The overall structure object as shown in Figure 1 is the high-level container object, and its constituent objects are various component unit objects and load objects. The component element object as a constituent object also includes constituent objects such as deformation matrix, stiffness matrix, and so on. Therefore, the component unit object is a sublevel container object [10].

#### 2.1.2. Dynamic Behavior Analysis

Dynamic behavior describes the sequence of legal states of objects in the software. The dynamic behavior of objects is usually represented by the dynamic model, which includes two aspects: one is the evolution of a single object's own life cycle; the other is the message passing and collaboration between objects in the whole object software.

The other is the message passing and collaboration between objects in the whole object software. One is the possible state of the object in its life cycle; the second is the action to be performed when the state transition occurs. The effect of the action depends not only on the operation of the object but also on the state the object is in. The third is the event that causes the transition of an object from one state to another. The event is the condition that controls the state transition. These three parts together constitute the state transition diagram in the object's life cycle to describe the internal dynamic behavior. The dynamic behavior of an object can be inherited by its children through inheritance relationships. The child object state diagram is a refinement of the parent object state diagram. In an object software, objects work together through message passing. For each task in the software, there is a set of messaging and actions on a set of objects to complete the task. Each object software has a set of tasks to complete, and each character has a sequence of events to correspond to. Therefore, the dynamic nature of object collaboration in the software can be described by a set of event sequences [11, 12].

### 2.2. Static Elastoplastic Analysis Method

Static elastoplastic analysis method, also known as the pushover analysis method, is an equivalent nonlinear static method. It attempts to use the static method to approximate the dynamic response performance of the structure. Using this analysis method can be calculated from the linear elastic structure, yield until the limit state of collapse of the internal force and deformation, the position, and angle of plastic hinge and find out the weak links of the structure, usually draw enough than the structure static elasticity analysis and nonlinear dynamic time history analysis of more important information. The commonly used static elastoplastic analysis is based on the following basic assumptions: (1) The response of a structure (usually a multi-degree-of-freedom software) is mainly controlled by its first mode shape, so the original structure can be replaced by an equivalent single-degree-of-freedom software and (2) the deformation of the structure along the height direction is represented by the shape vector {Φ}, and the shape vector {Φ} remains unchanged throughout the seismic response process. According to these two basic assumptions, a multi-degree-of-freedom software can be equivalent to a single-degree-of-freedom software [13–15].

The dynamic differential equation of the MDOF software under earthquake action is as follows:

(1) $$\begin{array}{c} \left[M\right]\left\{\ddot{X}\left(t\right)\right\}+\left[C\right]\left\{\ddot{X}\left(t\right)\right\}+\left\{Q\right\}=‒\left[M\right]\left\{I\right\}{\ddot{X}}_{g}\left(t\right). \end{array}$$

In formula (1), [*M*], [*C*], and {*Q*} are the mass matrix, damping matrix, and restoring force vector of the structure, respectively. For a linearly elastic structure, {*Q*}=[*K*]{*X*(*t*)}, {*X*(*t*)}, $\left\{\dot{X}\left(t\right)\right\}$, $\left\{\ddot{X}\left(t\right)\right\}$ represents the relative displacement response vector, the relative velocity response vector, and the relative acceleration response vector of the structure with respect to the base, respectively, $\ddot{X}\left(t\right)$ represents the earthquake ground motion acceleration [8].

According to the assumption, the structure displacement vector {*X*(*t*)} can be expressed by the structure vertex displacement *X*_*t*_(*t*) and the shape vector {Φ} as follows:

(2) $$\begin{array}{c} \left\{X\left(t\right)\right\}=\left\{\Phi \right\}X_{t}\left(t\right). \end{array}$$

Substituting equations (2) into (1), we can obtain the following;

(3) $$\begin{array}{c} \left[M\right]\left\{\Phi \right\}\left\{{\ddot{X}}_{t}\left(t\right)\right\}+\left[C\right]\left\{\Phi \right\}\left\{{\ddot{X}}_{t}\left(t\right)\right\}+\left\{Q\right\}=-\left[M\right]\left\{I\right\}{\ddot{X}}_{g}\left(t\right). \end{array}$$

The equivalent displacement of the equivalent single-degree-of-freedom software (SDOF) of the MDOF software is defined as *X*^*∗*^(*t*):

(4) $$\begin{array}{c} X^{*}\left(t\right)=\frac{{\left\{\Phi \right\}}^{T}\left[M\right]\left\{\Phi \right\}}{{\left\{\Phi \right\}}^{T}\left[M\right]\left\{I\right\}}X_{t}\left(t\right). \end{array}$$

That is,

(5) $$\begin{array}{c} X_{t}\left(t\right)=\frac{{\left\{\Phi \right\}}^{T}\left[M\right]\left\{I\right\}}{{\left\{\Phi \right\}}^{T}\left[M\right]\left\{\Phi \right\}}X^{*}\left(t\right). \end{array}$$

Substituting equations (5) into (3) and multiplying both sides of the equation by {Φ}^*T*^ to obtain the following:

(6) $$\begin{array}{c} M^{*}{\ddot{X}}^{*}\left(t\right)+C^{*}{\dot{X}}^{*}\left(t\right)+Q^{*}=-M^{*}{\ddot{X}}_{g}\left(t\right). \end{array}$$

In formula (6), *M*^*∗*^, *C*^*∗*^, and *Q*^*∗*^, respectively, represent the equivalent mass, equivalent damping, and equivalent restoring force of the equivalent single-degree-of-freedom software. The specific calculation expressions are as follows:

(7) $$\begin{array}{c} M^{*}={\left\{\Phi \right\}}^{T}\left[M\right]\left\{I\right\}. \end{array}$$

(8) $$\begin{array}{c} C^{*}={\left\{\Phi \right\}}^{T}\left[C\right]\left\{\Phi \right\}\frac{{\left\{\Phi \right\}}^{T}\left[M\right]\left\{I\right\}}{{\left\{\Phi \right\}}^{T}\left[M\right]\left\{\Phi \right\}}. \end{array}$$

(9) Q=∗ΦTQ.

In practice, the first mode vector is generally used as the shape vector. Obviously, this is only true when the structural response is controlled by the first mode. If the shape vector {Φ} is known, the force-deformation relationship of the equivalent single-degree-of-freedom software can be converted from the simplified force-deformation relationship of the original structure obtained by the pushover analysis method. The bottom shear at the yield point *Q*_*y*_ and the peak displacement *X*_*t*,*y*_ are obtained from the original structure. According to equations (4) and (9), the equivalent bottom shear *Q*_*y*_^*∗*^ and the peak displacement *X*_*y*_^*∗*^ at the yield point of the equivalent single-degree-of-freedom software can be obtained, namely,

(10) $$\begin{array}{c} X_{y}^{*}=\frac{{\left\{\Phi \right\}}^{T}\left[M\right]\left\{\Phi \right\}}{{\left\{\Phi \right\}}^{T}\left[M\right]\left\{I\right\}}X_{t,y}, \end{array}$$

(11) $$\begin{array}{c} Q_{y}^{*}={\left\{\Phi \right\}}^{T}Q_{y}. \end{array}$$

### 2.3. Energy-Based Modal Pushover Method

The advantage of modal pushover analysis (MPA) is that it can make full use of the existing single mode pushover analysis of the program to complete the analysis, and the accuracy of the analysis results can be greatly improved, which makes it highly applicable. In the process of MPA analysis, it is necessary to draw the base shear-to-reference point displacement curves of multiple modes and convert them into capability spectrum curves, so as to obtain the equivalent nonlinear software characteristics or to calculate the seismic response superimposed with the demand spectrum. From the above analysis, it can be seen that the fundamental reason for the deviation of the results of MPA analysis is that the local quantity, the displacement of a fixed reference point, is used to describe the pushover curve. Therefore, the improvement should start with the selection of reference displacement. According to the theory of structural analysis, the energy of a structure is an integral quantity, which includes the functions of each part of the structure. Therefore, based on the energy principle, this paper proposes to directly use the relationship between the energy increment and the displacement increment of each part of the structure to establish the capability spectrum curve for pushover analysis [16]. The following is a theoretical derivation of this method.

The energy balance equation of linear elastic single-degree-of-freedom software under earthquake action is as follows:

(12) $$\begin{array}{c} \int \left(m\ddot{u}+c\dot{u}+ku\right)\text{d}u=-\int m{\ddot{u}}_{g}\left(t\right)\text{d}u\\ \frac{1}{2}{\dot{u}}^{2}+2\zeta \omega \int \dot{u}\text{d}u+\frac{1}{2}\omega^{2}u^{2}=-\int {\ddot{u}}_{g}\left(t\right)\text{d}u. \end{array}$$

For a multi-degree-of-freedom software, the energy balance equation is as follows:

(13) $$\begin{array}{c} \int {\left\{\text{d}U\right\}}^{T}\left(\left[M\right]\left\{\ddot{U}\right\}+\left[C\right]\left\{\dot{U}\right\}+\left[K\right]\left\{U\right\}\right)=-\int \int {\left\{\text{d}U\right\}}^{T}\left[M\right]\left\{1\right\}{\ddot{u}}_{g}\left(t\right)=\int {\left\{\text{d}U\right\}}^{T}\left\{P_{eff}\left(t\right)\right\}. \end{array}$$

After simplification and consolidation, the following can be obtained:

(14) $$\begin{array}{c} \sum_{i=1}^{n}\left(\frac{1}{2}M_{i}{\dot{q}}_{i}^{2}\right)+\sum_{i=1}^{n}\int C_{i}{\dot{q}}_{i}\text{d}q_{i}+\sum_{i=1}^{n}\left(\frac{1}{2}K_{i}q_{i}^{2}\right)=-\sum_{i=1}^{n}\int {\ddot{u}}_{g}\left(t\right)L_{i}\text{d}q. \end{array}$$

Equation (14) can be considered as the superposition of the energy balance equations of *n* single-degree-of-freedom linear software *s*, namely,

(15) $$\begin{array}{c} \frac{1}{2}M_{i}{\dot{q}}_{i}^{2}+\int C_{i}{\dot{q}}_{i}\text{d}q_{i}+\frac{1}{2}K_{i}q_{i}^{2}=-\int {\ddot{u}}_{g}\left(t\right)L_{i}\text{d}q_{i}\;\left(i=1,\ldots ,n\right). \end{array}$$

We substitute (15) into formula (14)to obtain the following:

(16) $$\begin{array}{c} \sum_{i=1}^{n}\left(\frac{1}{2}M_{i}{\dot{q}}_{i}^{2}\right)+\sum_{i=1}^{n}\int C_{i}{\dot{q}}_{i}\text{d}q_{i}+\sum_{i=1}^{n}\left(\frac{1}{2}K_{i}q_{i}^{2}\right)=-\int {\ddot{u}}_{g}\left(t\right)L_{k}\text{d}q_{k}. \end{array}$$

When *i* ≠ *k*, there is *q*_*i*_=0, then (16) becomes as follows:

(17) $$\begin{array}{c} \frac{1}{2}M_{k}{\dot{q}}_{k}^{2}+\int C_{k}{\dot{q}}_{k}\text{d}q_{k}+\frac{1}{2}K_{k}q_{k}^{2}=-\int {\ddot{u}}_{g}\left(t\right)L_{k}\text{d}q_{k}. \end{array}$$

Formula (17)is the same as the energy balance equation (equation (15)) of the KTH degree of freedom software. Simplify to equation (16).

Equations (16) and (17) indicate that the total input energy of the structure is all converted into the total energy of the KTH order single-degree-of-freedom software under the action of the KTH effective modal force [17, 18].

## 3. Result Analysis

### 3.1. Probability Pushover Analysis

**Figure 3:**
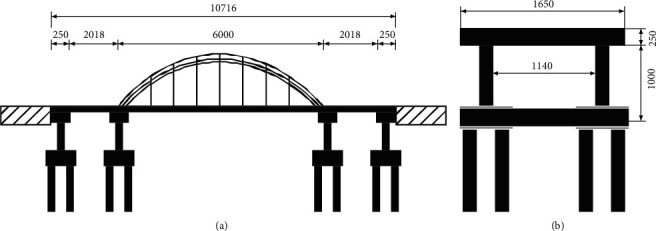
Bridge structure brie diagram of a city (unit:cm). (a) General layout. (b) Structural drawing of pier.

The example bridge is shown in Figure 3(a) of a city bridge, whose bridge layout adopts 20 m hollow slabs +60 m simply supported concrete-filled steel tube tie arch ++20 m hollow slabs, with a total length of 107.16 m and a clear width of bridge deck of 2 × 2.0 m sidewalk + 8M lane. The tie arch is a 1–60 m downhole simply supported concrete-filled steel tube arch. The theoretical span of the arch rib is 60 m, the calculated height of the arch is 12 m, and the ratio of the span of the arch is 1/5. The theoretical arch axis equation is *Y* = 4*X*/5 − *xz*/75(coordinate origin is the theoretical arching point). The bridge deck structure adopts the vertical and horizontal beam software and the integral bridge deck to improve the overall stiffness of the structure. The two ends of the side span are prestressed concrete hollow slabs. The piers adopt circular section column piers with cover beams, and the height distribution of the two piers is 10.0 m and 7.0 m. The section diameter of the pier is 200 cm, and the height of the cover beam is 150 cm, as shown in Figure 3(b). In order to facilitate the connection with the side span hollow plate, the cover beam is raised on the side of the hollow plate to form an L-shaped section, but several false joints are set in the raised part to prevent the joint force with the cover beam. The pier foundation adopts eight 120 cm bored cast-in-place piles, most of which are embedded in the rock stratum. The main pier cover beam and pier column are made of C40 concrete. The bridge pier section is reinforced with 40 25 mm HRB335 steel bars. The stirrups are Grade I steel bars with a stirrup ratio of 0.003. The basic intensity of the bridge site is 7 degrees, the design basic acceleration (i.e., the acceleration with the probability of surpassing to% in so year) is O.Lg, the foundation of the engineering site is class *B*, and the characteristic period is 0.4 s.

**Figure 4:**
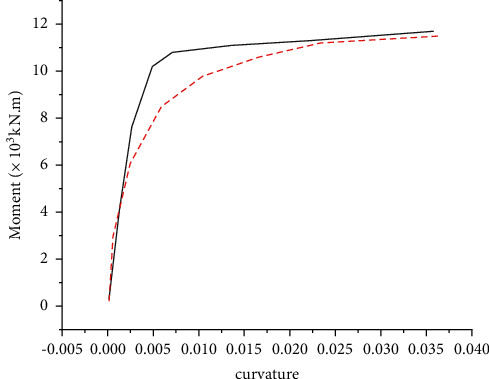
Bending moment-curvature curve of pier section.

First, the moment-curvature analysis of bridge pier is carried out. Because the counterforce of the fulcrum passed down by the superstructure is far greater than the gravity of the pier itself, the axial force at the top and bottom of the pier is approximately equal. Therefore, according to the geometry of the pier, the reinforcement situation, and the action of the axial force, the bending moment curvature curve of the pier is as shown in Figure 4.

**Figure 5:**
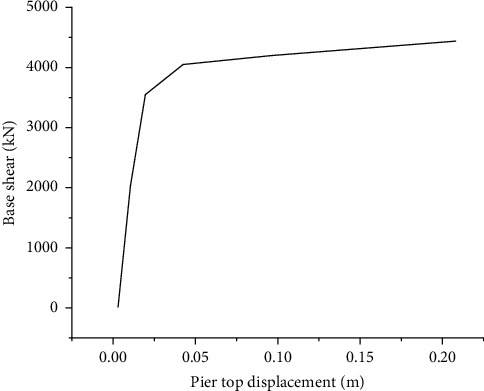
Pushover curve of bridge pier.

The plastic hinge characteristics can be defined according to the above bending moment-curvature. For the piers analyzed, the positions of plastic hinge are pier bottom and pier top. Therefore, plastic hinges were introduced at the pier top and pier bottom to conduct pushover analysis, and the pushover curve was obtained, as shown in Figure 5.

### 3.2. Seismic Reliability in the 50-Year Base Period

According to the seismic risk curve, the probability distribution of random earthquake action in the 50-year base period can be obtained, as follows:

(18) $$\begin{array}{c} F_{A}\left(A\right)=\exp\left[-0.1054{\left(\frac{A}{A_{10}}\right)}^{-K}\right], \end{array}$$

where *A*_10_ is the acceleration exceeding 10% of the probability in the 50-year base period, that is, the basic acceleration specified in the zoning map of ground motion parameters in China. *K* is the shape coefficient, and the national average value is 2.35, which can also be taken separately according to the seismicity of different regions. Here, we take the national average for analysis. The strength reduction coefficient model mentioned above is still adopted, and the probability distribution function of ductility demand *μ* under random earthquake action in 50 reference period can be obtained as follows:

(19) $$\begin{array}{c} F_{\mu s}\left(\mu \right)=\exp\left[-0.1054{\left(-\frac{A_{y}{\left[c\left(\mu -1\right)+1\right]}^{1/c}}{A_{10}}\right)}^{-k}\right]. \end{array}$$

By substituting (18) into (20), the failure probability in the base period of so year can be obtained. For the yield limit state, the failure probability is as follows:

(20) $$\begin{array}{c} p_{f0}=P\left(A_{e}\geq A_{y}\right)=1-F_{A}\left(A_{y}\right)=1-\text{EXP}\left[-0.1054{\left(\frac{A_{y}}{A_{10}}\right)}^{-k}\right]. \end{array}$$

**Figure 6:**
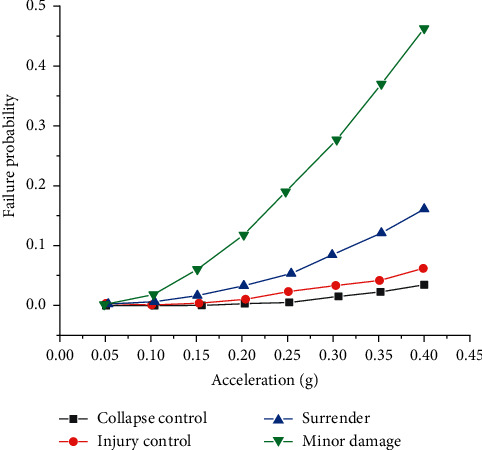
Failure probability of each limit state of the structure under different fundamental accelerations in a 50-year base period.

After obtaining the conditional failure probabilities of all discretized pushover curves, the total failure probabilities under a given earthquake can be obtained by substituting them into equations (7)–(10). Thus, the failure probabilities of the structure in each limit state under earthquake action with different basic accelerations are obtained as shown in Table 1 and Figure 6.

Through the analysis of an engineering example, the application of the proposed method in the reliability analysis of seismic performance of bridge structures is illustrated in detail. The calculation process and the obtained results show that the failure probability of each limit state of the structure is less than 0.5 under the earthquake action of different basic accelerations from 0 to 0.4 g. The proposed method can not only be used to analyze the reliability of seismic performance based on the strength failure but can also be used to analyze the reliability of the inelastic seismic performance of structures under large earthquakes conveniently. Using this method, the failure probability of the bridge structure under the seismic level can be calculated directly, and if the economic losses under different failure states of the bridge structure are given, the expected economic losses caused by seismic disasters can also be estimated during the design life. All these provide a scientific basis for decision makers. Seismic performance of the structure reliability analysis method for avoiding the traditional structural aseismic reliability analysis of large-scale Monte Carlo sampling simulation, greatly reducing the workload of calculation (especially for nonlinear structure), is based on probability performance based seismic design and evaluation method is a simple and effective method.

## 4. Conclusions

The basic theory of bridge structure analysis and some solutions of object-oriented technology are analyzed by software. The method of structural analysis software calculation using object-oriented programming technology has greatly improved the software extensibility and code reusability. The pushover method based on energy is proposed. The seismic performance of a continuous rigid frame bridge is analyzed by using the proposed method. The results show that the proposed method has good adaptability, simple calculation, and strong adaptability and can be applied to the nonlinear seismic performance analysis of more complex structures. At the same time, the digital level of linear engineering management, such as bridges, is improved to provide technical support for the quality construction of the project.

## Figures and Tables

**Table 1:** Failure probabilities of bridge piers in each limit state under seismic action in a 50-year base period.

Limit state *A*_10_	0.1 g	0.15 g	0.2 g	0.3 g	0.4 g
Yield	0.0241	0.0612	0.1163	0.2714	0.4566
Minor damage	0.0068	0.0176	0.0344	0.0863	0.1616
Damage control	0.0024	0.0062	0.0122	0.0312	0.0602
Collapse control	0.0014	0.0036	0.0070	0.0179	0.0347

## Data Availability

The data used to support the findings of this study are available from the corresponding author upon request.
